# Use and misuse of biomarkers and the role of D-dimer and C-reactive protein in the management of COVID-19: A post-hoc analysis of a prospective cohort study

**DOI:** 10.6061/clinics/2021/e3547

**Published:** 2021-11-24

**Authors:** Fabio Augusto Rodrigues Gonçalves, Bruno Adler Maccagnan Pinheiro Besen, Clarice Antunes de Lima, Aline Pivetta Corá, Antônio José Rodrigues Pereira, Sandro Félix Perazzio, Christiane Pereira Gouvea, Luiz Augusto Marcondes Fonseca, Evelinda Marramon Trindade, Nairo Massakazu Sumita, Alberto José da Silva Duarte, Arnaldo Lichtenstein, HCFMUSP COVID-19 Study Group, Eloisa Bonfa, Edivaldo M. Utiyama, Aluisio C. Segurado, Beatriz Perondi, Anna Miethke-Morais, Amanda C. Montal, Leila Harima, Solange R. G. Fusco, Marjorie F. Silva, Marcelo C. Rocha, Izabel Marcilio, Izabel Cristina Rios, Fabiane Yumi Ogihara Kawano, Maria Amélia de Jesus, Ésper George Kallas, Carolina Carmo, Clarice Tanaka, Heraldo Possolo de Souza, Julio F. M. Marchini, Carlos Carvalho, Juliana C. Ferreira, Anna Sara Shafferman Levin, Maura Salaroli Oliveira, Thaís Guimarães, Carolina dos Santos Lázari, Ester Sabino, Marcello M. C. Magri, Tarcisio E. P. Barros-Filho, Maria Cristina Peres Braido Francisco, Silvia F. Costa

**Affiliations:** I Laboratorio de Cirurgia Cardiovascular e Fisiopatologia da Circulacao (LIM11), Faculdade de Medicina FMUSP, Universidade de Sao Paulo, Sao Paulo, SP, BR.; II Unidade de Terapia Intensiva, Disciplina de Emergencias Clinicas, Faculdade de Medicina FMUSP, Universidade de Sao Paulo, Sao Paulo, SP, BR.; III Departamento de Medicina Interna, Instituto Central, Hospital das Clinicas HCFMUSP, Faculdade de Medicina, Universidade de Sao Paulo, Sao Paulo, SP, BR.; IV Divisao de Laboratorio Central, Hospital das Clinicas HCFMUSP, Faculdade de Medicina, Universidade de Sao Paulo, Sao Paulo, SP, BR.; V Superintendencia, Hospital das Clinicas HCFMUSP, Faculdade de Medicina, Universidade de Sao Paulo, Sao Paulo, SP, BR.; VI Servico de Imunologia Clinica e Alergia, Hospital das Clinicas HCFMUSP, Faculdade de Medicina, Universidade de Sao Paulo, Sao Paulo, SP, BR.; VII Nucleo de Avaliacao de Tecnologia em Saude, Hospital das Clinicas HCFMUSP, Faculdade de Medicina, Universidade de Sao Paulo, Sao Paulo, SP, BR.; VIII Laboratorio de Dermatologia e Imunodeficiencias (LIM56), Faculdade de Medicina FMUSP, Universidade de Sao Paulo, Sao Paulo, SP, BR.; Hospital das Clinicas HCFMUSP, Faculdade de Medicina, Universidade de Sao Paulo, Sao Paulo, SP, BR.

**Keywords:** COVID-19, Biomarkers, Cohort Studies, Venous Thromboembolism, Health Care Costs

## Abstract

**OBJECTIVE:**

Coronavirus disease 2019 (COVID-19) is associated with high mortality among hospitalized patients and incurs high costs. Severe acute respiratory syndrome coronavirus 2 infection can trigger both inflammatory and thrombotic processes, and these complications can lead to a poorer prognosis. This study aimed to evaluate the association and temporal trends of D-dimer and C-reactive protein (CRP) levels with the incidence of venous thromboembolism (VTE), hospital mortality, and costs among inpatients with COVID-19.

**METHODS:**

Data were extracted from electronic patient records and laboratory databases. Crude and adjusted associations for age, sex, number of comorbidities, Sequential Organ Failure Assessment score at admission, and D-dimer or CRP logistic regression models were used to evaluate associations.

**RESULTS:**

Between March and June 2020, COVID-19 was documented in 3,254 inpatients. The D-dimer level ≥4,000 ng/mL fibrinogen equivalent unit (FEU) mortality odds ratio (OR) was 4.48 (adjusted OR: 1.97). The CRP level ≥220 mg/dL OR for death was 7.73 (adjusted OR: 3.93). The D-dimer level ≥4,000 ng/mL FEU VTE OR was 3.96 (adjusted OR: 3.26). The CRP level ≥220 mg/dL OR for VTE was 2.71 (adjusted OR: 1.92). All these analyses were statistically significant (*p*<0.001). Stratified hospital costs demonstrated a dose-response pattern. Adjusted D-dimer and CRP levels were associated with higher mortality and doubled hospital costs. In the first week, elevated D-dimer levels predicted VTE occurrence and systemic inflammatory harm, while CRP was a hospital mortality predictor.

**CONCLUSION:**

D-dimer and CRP levels were associated with higher hospital mortality and a higher incidence of VTE. D-dimer was more strongly associated with VTE, although its discriminative ability was poor, while CRP was a stronger predictor of hospital mortality. Their use outside the usual indications should not be modified and should be discouraged.

## INTRODUCTION

Coronavirus disease 2019 (COVID-19) is a disease associated with high mortality among hospitalized patients and incurs high hospitalization costs, long-term consequences, and increased burden on the healthcare system and society (1). Several studies have indicated that severe acute respiratory syndrome coronavirus 2 (SARS-CoV-2) infection triggers both inflammatory and thrombotic processes (2). Previous studies have already reported on prognostic factors, coagulation disorders, and thrombotic events associated with the severe pattern of COVID-19 and poor prognosis of the disease (3-7).

Given the hypothesized and posteriorly confirmed hypercoagulability milieu (8), some authors suggest that a routine evaluation of the levels of D-dimer and other biomarkers of hypercoagulability should be conducted; alternatively, they recommend either the initiation of a routine full-dose anticoagulation or an extended-dose prophylactic anticoagulation (9). Of note, D-dimer levels have been validated in clinical practice for two main purposes: first, to evaluate patients with suspected venous thromboembolism (VTE) at a low or intermediate clinical probability, as assessed by validated scales; second, as part of the disseminated intravascular coagulation (DIC) score proposed by the *International Society for Thrombosis and Hemostasis* (10). Its use as a biomarker to guide therapy has not yet been validated in inflammatory conditions.

The use of prophylactic anticoagulation can improve the outcome of COVID-19, reiterating the established standard of care for critically hospitalized patients admitted with infection and reduced mobility (9). Clinical trials (e.g., ProCESS) have assessed the relative benefits and risks of alternative anticoagulation strategies (11). Some recent clinical trials demonstrated that, compared with standard-dose prophylaxis, neither an intermediate-dose prophylactic anticoagulation nor full-dose anticoagulation has been associated with improved outcomes (12,13). However, other trials have demonstrated the benefit of full-dose anticoagulation in severe, but not critical COVID-19, regardless of D-dimer levels (14,15). Some of these trials used and recommended D-dimer levels as prognostic enrichment biomarkers, whereas others did not.

In this scenario, the rational use of laboratory tests, specifically D-dimer levels, is of utmost importance. Routine use of tests out of its original purpose, based on pathophysiological theories, may lead to downstream-unintended consequences, such as shortages of consumables and increased healthcare costs. Furthermore, full dose anticoagulation implies a higher risk of bleeding if the D-dimer results guide the initiation of treatment.

In this context of uncertainty regarding the clinical usefulness and risks for routine monitoring of D-dimer levels, we designed this post-hoc analysis of this local hospitalized cohort to study the association between D-dimer levels and hospital mortality and the incidence of VTE, accounting for the link between inflammation and thrombosis. Our objective was to describe and to evaluate both the prognostic impact of D-dimer levels and its diagnostic utility in the context of a highly inflammatory condition, such as COVID-19, along with their associated impact on healthcare costs.

## MATERIALS AND METHODS

This is a post-hoc analysis of a prospective observational cohort economic study undertaken at the Instituto Central Hospital das Clinicas (HCFMUSP), Faculdade de Medicina, Universidade de Sao Paulo, SP, Brazil (ICHC). Patients from the cohort were admitted from March 30 to June 30, 2020 and followed until discharge, death, external transfer, or up to August 25, 2020. Because of the observational nature of this post-hoc analysis, the institutional review board waived the requirement for patients’ informed consent (CAPPESQ: #4.107.580). This report complies with the Strengthening the Reporting of Observational Studies in Epidemiology statement (16).

### Setting and participants

Participants were included if they were admitted with suspected or polymerase chain reaction (PCR)-confirmed SARS-CoV-2 infection. The exclusion criteria were age <18 years and absence of a D-dimer test. The ICHC (a hospital with 900 beds; 150 intensive care unit [ICU] beds extended to 300 ICU beds at the peak of the pandemic) was transformed into an exclusive COVID-19 hospital because of cross-contamination concerns. Further characteristics have been described previously in other manuscripts (1,17,18).

On hospital admission, the hospital protocol suggested ordering D-dimer levels. Further VTE diagnostic radiologic tests could be ordered at the clinician’s discretion but not recommended as routine, based solely on D-dimer levels. Other recommended laboratory tests included those commonly ordered for pneumonia risk stratification, organ dysfunction, and electrolyte disturbance evaluation. Further tests were requested at the clinician’s discretion and on a hypothesis-driven basis. The ICHC thromboprophylaxis protocol included both the use of unfractionated heparin (UFH) or low-molecular-weight heparin (LMWH, enoxaparin). A weight-based dose was recommended instead of routine low-dose prophylaxis or full-dose anticoagulation. Both LMWH and UFH at full dose were administered at the clinician discretion in cases of suspected or confirmed VTE or for other required indications. Intravenous UFH at an intermediate dose was used to increase filter patency during continuous dialysis or hemofiltration, with the specific aim of avoiding full-dose anticoagulation (19). This ICHC protocol also recommends full-dose intravenous UFH when there are contraindications to LMWH.

### Data collection and variables

The prospective clinical data were collected from *the Electronic Health Records* of ICHC, including sex, age, COVID-19 confirmation, underlying medical conditions, procedures (mechanical ventilation and dialysis), prescribed tests and drugs (heparin, enoxaparin, vasoactive drugs, among others), patient itinerary (length of stay in the emergency department [ED], hospital wards, and ICUs), and primary outcome (discharge, death, or transfer). We obtained laboratory test results from the hospital laboratory database. We calculated a modified Sequential Organ Failure Assessment Score (mSOFA, defined without the neurologic component) from the collected variables to stratify risk adjustment and statistics (20).

Laboratory parameters were categorized as normal (N) or altered (A), according to the established assay cut-off method. Standard measurement methods established at the Central Laboratory Division of HCFMUSP were chemiluminescence for COVID-19 serology on a Liaison XL analyzer (DiaSorin S.p.A., Saluggia, Italy), PCR for confirmation on Abbott m200RT (Abbott Laboratories, Chicago, IL, USA), and quantitative immunoturbidimetric assay for D-dimer on an ACL TOP 750 automated coagulation analyzer (IL, Werfen, MA, USA), immunoturbidimetric assay for C-reactive protein (CRP) on a Cobas c702 analyzer (Roche Diagnostics, Basel, Switzerland), and electrochemiluminescence for ferritin on a Cobas e602 analyzer (Roche Diagnostics, Basel, Switzerland).

We first screened clinician escalation to full dose anticoagulation according to the prescribed dose of anticoagulants: ≥80 mg/day of enoxaparin or ≥100 mg/day in obese patients (body mass index ≥30 kg/m^2^); any use of intravenous UFH in non-dialysis patients; or use of intravenous UFH in patients who underwent dialysis associated with ≥2.0 at the test result ratio (R) for activated partial thromboplastin time. These criteria need to be fulfilled for two or more consecutive days. After this screening, we evaluated imaging test results (computed tomography pulmonary angiography and venous Doppler ultrasonography) at the patient’s medical records to confirm VTE. We defined confirmed VTE as patients who underwent a full-dose anticoagulation and underwent a confirmatory test.

### Economic analysis

The economic analysis was performed from the clinical hospital perspective using mixed and micro-costing methodology (21,22). The resources used by each patient were identified, quantified, and valued to ascertain and to describe the admissions’ individual costs. Direct and fixed costs are also included. Direct cost subcategories include micro costing for admissions, and individual admission costs included drugs, laboratory tests, radiologic examinations, blood components, and nutrition requirements. Cost values observed at the ICHC’`s 2020 resource bulk buys were used to build these micro direct costs. Direct costs of hospital supplies, days in ER, wards and ICUs, and service fixed costs (laundry, administration, maintenance contracts, and general services) from each sector were apportioned by patient-bed-day to estimate a general daily cost for each specific sector.

Other fixed direct costs (such as human resources, medical staff, and non-medical staff who are paid independent of production) and indirect costs that were not related to the patient’s hospital admission (e.g., outpatient hospital visits, patient transportation, and others) or from the hospital’s perspective (such as productivity losses) were not included in this analysis.

The total admission cost for each patient was estimated by the daily costs according to the patient's itinerary in the ED, wards, and ICUs added to the direct costs measured by the direct consumption observed during the study period. Hospital resource used costs were ascertained by the patient and classified by primary outcome and by relevant strata for discussion.

The financial data were collected in Brazilian currency (Reais, in 2020) and converted into US dollars according to the purchasing power parity value (US$ 2.362, in 2020) (23).

### Statistical analyses

There were no a priori sample size calculations. We used a convenience sample of all patients who met the inclusion and exclusion criteria during the study period. In the descriptive analysis, continuous variables were expressed as means±standard deviations or confidence intervals and categorical variables were presented as counted cases or their respective absolute and relative distributions. Kaplan-Meier survival curves were used to describe the temporal distribution and incidence density of events, outcomes, and associated test results. The total cost of each patient admission was analyzed and described for the study period. The impact of different variables on hospital cost was assessed according to the average cost for each patient subgroup. Each subgroup’s average and dispersion costs were estimated by adding the related number and costs of admissions divided by the respective number of experienced patient-days during the study period.

To evaluate the association between D-dimer and CRP levels with hospital mortality and the development of VTE, we developed sequential logistic regression models adjusting for confounding factors. We used the worst values from these tests throughout the hospital stay. These models included: [1] crude analysis; [2] adjusted for age and sex; [3] adjusted for [2] and the number of comorbidities; [4] adjusted for [3] and the mSOFA; and [5] adjusted for item [4] variables and either D-dimer or CRP (according to each model) to ascertain the D-dimer association with inflammation and thrombosis or CRP association with the outcomes. For these analyses, we dichotomized D-dimer and CRP levels, based on the Youden index from the area under the receiver-operating characteristic curves (AU-ROCs).

Continuous and categorical variables were compared among subgroups using the Wilcoxon rank sum test and Fisher’s exact test for counted data and independent samples. Univariate and multiple linear regression analyses were performed to identify predictors for COVID-19 hospitalization outcomes and cost. The candidate variables were age, sex, comorbidities, procedures, and mortality. A generalized linear model under gamma distribution was fitted to ascertain the impact magnitude of the variables on the total costs. In all analyses, cases with missing data were not imputed but were excluded from the specific analysis. Statistical analyses were performed using R (v4.1.0) (24-26). Statistical significance was set at *p*<0.05.

## RESULTS

### Sample characterization

Between March 30, 2020 and June 30, 2020, 3,254 admissions of patients with suspected or confirmed COVID-19 were recorded (54.5% males, with an overall mean [±standard deviation] age of 57.8 [±17.7 years]). COVID-19 real-time PCR was confirmed for a total of 2,512 patients (77.2%), and the remaining patients were treated for presumed infection based on clinical and/or radiologic findings.

Overall, 51.7% were admitted to the ICU during hospital stay: 62% of patients were discharged alive, 939 (28.9%) died during admission, and 278 (8.5%) were transferred to other facilities.

Only 376 (11.6%) patients had no comorbidities. The remaining patients had 1 (23.2%), 2 or 3 (40%), or >3 (13.9%) comorbidities. The most frequent comorbidities were hypertension (48.1%), diabetes mellitus (30.5%), previous or current smoking (24.6%), and obesity (23%).

During the study period, amid COVID-19 confirmed patients, there were 2,361,677 test items; further, D-dimer results were available for 2,478 patients from the database, where 55% were males and had significantly more events, that is, 33% died, in comparison with 24% deceased females tested (Table 1). Creatinine test results were available for 2,472 patients of 2,478 with D-dimer test results from the database. Ferritin test results were available for 780, and 2,415 of 2,478 patients with D-dimer results from the database had CRP test results available.

### D-dimer and CRP examination characteristics and univariate associations

**Figure 1:**
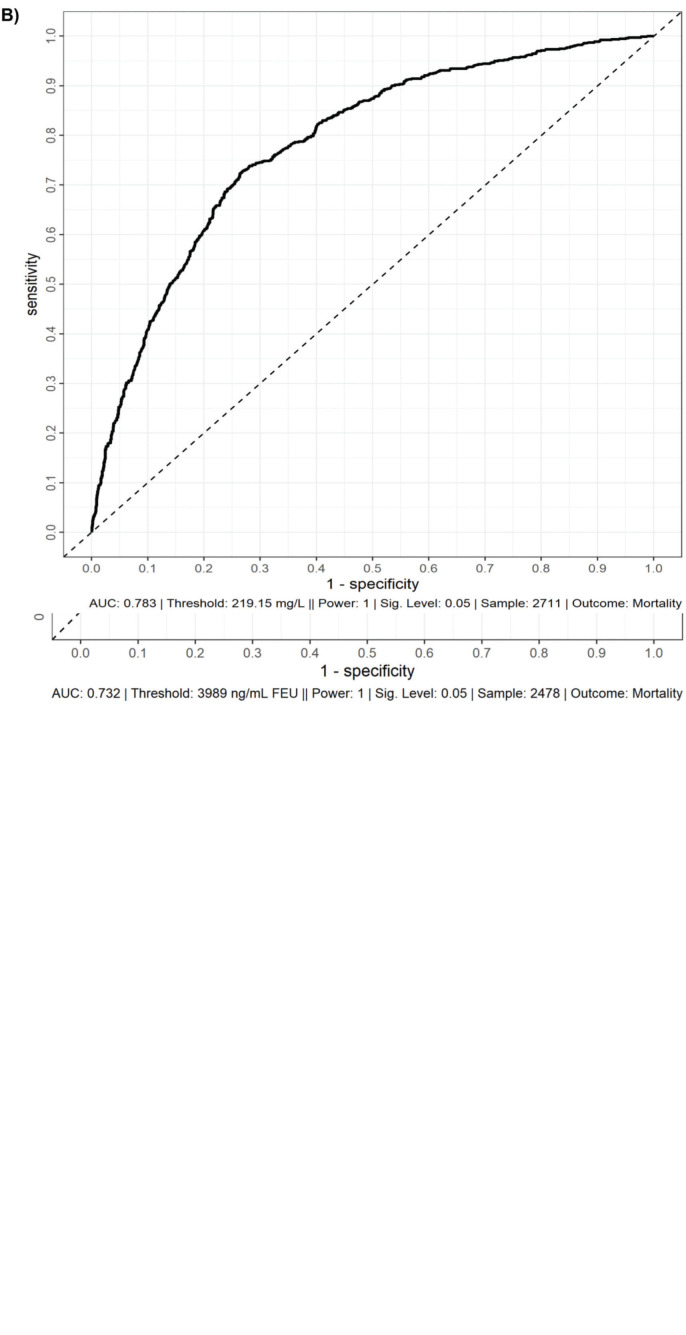
ROC curves of D-Dimer (A) and C-reactive protein (B) with hospital mortality as the outcome. These ROC curves demonstrate the discrimination of D-dimer (A) and C-reactive protein (B) for hospital mortality. ROC, receiver operating characteristic curve; AUC, area under the curve; FEU, fibrinogen equivalent units.

**Figure 2:**
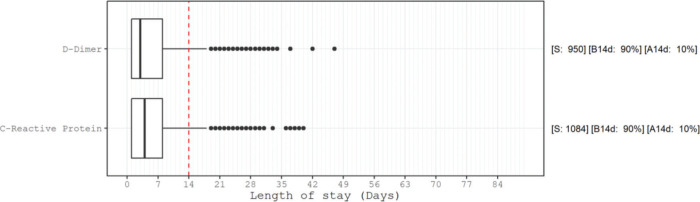
Box plot of worst test result temporal distribution, sample size, and proportion of the altered results. Distribution of the day of the worst altered (according to the cut-off from Figure 1) results in the D-dimer test and C-reactive protein measurements. Note that 75% of these worst results occurred up to the 8^th^ day of hospitalization. S: number of patients with altered results; B14d: proportion of altered results before 14 days of hospitalization; A14d: proportion of altered results after 14 days of hospitalization.

**Figure 3:**
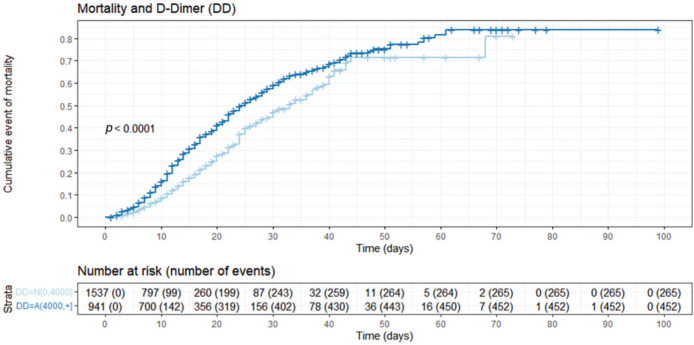
Nelson-Aalen cumulative mortality plot stratified by D-dimer levels. This figure describes the cumulative mortality stratified by the D-dimer cut-off (greater or lower than 4,000 ng/mL FEU), with higher cumulative mortality observed in patients with high D-dimer levels. DD=N[0, 4,000]: D-dimer levels up to 4,000 ng/mL FEU; DD=A(4,000, +): D-dimer levels greater than 4,000 ng/mL FEU.

**Figure 4:**
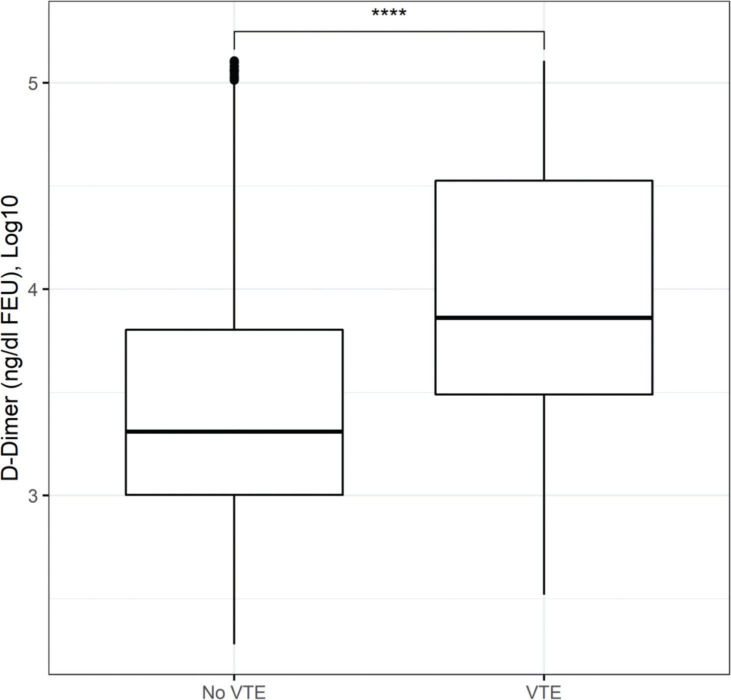
Box plot of Log_10_ D-Dimer stratified by the occurrence of venous thromboembolism (VTE). This box plot shows a large overlap of D-dimer levels among patients with or without VTE in this cohort, suggesting a low discriminative ability in this population.

Figure 1 presents the AU-ROCs for both D-dimer and CRP in relation to hospital mortality outcomes. Both present some discriminatory prediction ability for hospital mortality, revealed by an estimated average AU-ROC of 0.75. The temporal distribution of most abnormal values test results occurred predominantly within the first week of admission (Figure 2). The associated D-dimer test result threshold was 4,000 ng/mL fibrinogen equivalent unit (FEU) (eight times the expected 500 ng/mL FEU upper limit of the normal cut-off). Patients whose D-dimer test results were greater than the ≥4,000 ng/mL FEU estimated threshold had significantly higher hospital mortality (Table 2, Figures 1 and 2). Almost half of the patients with D-dimer levels of ≥4,000 ng/mL FEU threshold [more than eight times the upper limit of normal (UNL)] died in the hospital (Figure 3). In addition, the most severe D-dimer level at any admission day showed a dose-response relationship with mortality, increasing from 8% in patients with results less than two times the ULN, 18% with less than four times the ULN, 27% with less than eight times the ULN, and up to 63% mortality among patients with D-dimer levels ≥4,000 ng/mL FEU threshold (Figures 3 and 4, Table 4).

### Multivariable analysis

Table 2 presents crude and adjusted associations between D-dimer and CRP levels with hospital mortality. While both test results were associated with increased hospital mortality, the adjusted association was stronger for CRP (a biomarker of inflammation) than for D-dimer levels (a biomarker of thrombosis), even when adjusted for each other. After adjusting for age, sex (Model 1), comorbidities (Model 2), and mSOFA (Model 3), all results remained significantly associated with hospital mortality. When adjusted for confirmed VTE during hospitalization, the D-dimer and CRP levels maintain significant prediction power for hospital mortality (Model 5, Table 2).

For patients with confirmed VTE outcomes during hospitalization, Table 3 presents the crude and adjusted associations of D-dimer and CRP levels. D-dimer was more strongly associated with VTE occurrence than CRP, although both were VTE-associated, even when adjusted for age, sex, comorbidities, mSOFA, and other tests, such as CRP and D-dimer.

Moreover, the relationship between test results and mortality was ascertained using the D-dimer AUC-ROC threshold, which also delimited the other related inflammatory tests performed for those patients. It also reflects the mean levels and dispersion with a dose response pattern, as evidenced by the patients where they occurred. Categorized accordingly (Figure 4, Table 4), (i) for each outcome group, the D-dimer, CRP, and ferritin test result means were significantly different from their reference subgroups below its own AUC-ROC thresholds: D-dimer was 12-fold higher for females and 15-fold higher for males; the CRP means were 2 times higher for alive females and 1.5 times higher for alive males, with only an approximately 10% increase for those who died regardless of sex. Moreover, for deceased males, the mean ferritin test results (smaller sample) were 4 times higher; (ii) for within subgroups, there were only small differences and were significantly different between adults and older individuals aged >65 years.

Patients who used LMWH presented a significantly reduced mortality rate compared with those who used UFH (Table 4), indicating the influence of other comorbid conditions. Since the use of UFH thromboprophylaxis for dialysis patients is based on the ICHC protocol, we investigated their association with kidney dysfunction. Indeed, more patients with chronic kidney disease were administered therapeutic levels of anticoagulants, presented the highest D-dimer levels, and died (Figure 4). Although presenting less altered D-dimer test results, in acute kidney injury, patients also used anticoagulants at therapeutic levels and had a higher mortality rate than those for whom thromboprophylaxis was not escalated to therapeutic levels.

Table 5 (cost components) indicates the relevant directions for care resource planning. The D-dimer test was associated with the studied outcomes and modified healthcare resources required during hospital admissions for SARS-CoV-2 infection.

Indeed, alive patients with VTE events doubled their length of hospital stay, resulting in more total costs (2.5 times) compared with alive patients without VTE. Although deceased patients without VTE had an average length of hospital stay of only 20%, they also presented a significant 2.5 times the total costs. For patients with both events, the impact on admission healthcare resources required was increased to 3.4 times compared with alive patients without VTE.

VTE events triggered a >50% increase in wards, mechanical ventilation, parenteral nutrition, and staff cost components and doubled the intensity of use and costs for imaging examinations. In comparison, deceased patients required two times more dialysis and mechanical ventilation, regardless of the presence or absence of a VTE event. Overall, such features highlight the importance of accurate event prognosis for healthcare planning. Thus, in our study, the D-dimer test also had a role and impact on the intensity of resources required during hospital admissions for SARS-CoV-2 infection.

## DISCUSSION

In this analysis, among 2,478 patients with D-dimer test results, 255 (10.2%) had a confirmed VTE, and we observed an absolute 30% hospital mortality. D-dimer and CRP levels were frequently measured in patients with COVID-19 admitted during the first peak of the pandemic, with its worse altered values more frequently observed during the first week of hospital stay. Both elevated D-dimer and high CRP levels were associated with increased hospital mortality and a higher risk of VTE after adjusting for available confounders and for the known biological association between thrombosis and inflammation. Indeed, the inflammatory biomarker CRP was more strongly associated with hospital mortality than D-dimer levels, while D-dimer levels were more strongly associated with VTE than CRP. Finally, their association with outcomes was attenuated by each other, again reiterating the known link between inflammation and thrombosis in acute critical illness, including COVID-19.

The incidence of thrombosis in our cohort of hospitalized patients with COVID-19 was comparable to that in prior reports of hospitalized patients (11%-16%) who received prophylactic anticoagulation (10). This lower incidence than in quoted literature reports may be because of our stringent imaging evidence criteria for VTE confirmation. Furthermore, 300 patients received full-dose anticoagulation, without a confirmed VTE. This could include patients with prior atrial fibrillation or who for some reason were already on full-dose anticoagulation and those patients who had suspected VTE but without further confirmation. Finally, some patients may have been put under full-dose anticoagulation at the discretion of the clinician. Taken together, these results suggest that the incidence of VTE may not be as high as that suggested by previous reports.

D-dimer has been evaluated frequently in patients admitted because of COVID-19 as a therapeutic guide for choosing the anticoagulation strategy despite poor experimental evidence at the onset of the pandemic. However, this test is not indicated for this purpose in practice. The D-dimer assay was originally intended to exclude VTE associated with a low or moderate clinical probability of a thrombotic event, given its high negative predictive value. Among patients with COVID-19, other factors, such as inflammatory activity, hospitalization, associated infections, and previous comorbidities, may act as confounders. Figure 4 highlights this issue, with a large overlap of D-dimer levels among patients with and without VTE. As has been the best practice since the advent of routine D-dimer availability, its ability to diagnose VTE is quite poor, and our data reaffirm this, suggesting that the long-standing guidance of using it only as a rule-out test for patients with a low or moderate probability of VTE should be maintained.

Other aspects to be highlighted are the numerous methodologies and reagents available for D-dimer determination, each one with its respective sensitivity, specificity, and interferences, among which the most commonly described are the presence of a rheumatoid factor and heterophile antibodies (34-36).

We observed an association with higher hospital mortality at a cut-off of 8 times the normal upper limit for D-dimer (Figure 1). D-dimer levels were lower (1,617 ng/mL FEU [875-4,721]) among survivors than among deceased patients (6,282 ng/mL FEU [2,148-21,580]), as shown in Table 1. Figure 2 further demonstrates this association in a survival analysis framework. Furthermore, a dose-response relationship was observed according to the D-dimer cut-off: at 2 times the upper normal limit, the mortality rate was 8%; at 4 times the UNL, it was 18%; and at >8 times the UNL, it was 63%. This higher cut-off value has also been observed in other studies, ranging from 2,000 ng/mL FEU to 4,000 ng/mL FEU (10). Since the validated prognostic scores in COVID-19 did not include this D-dimer prognostic ability in their model, further adequately designed studies are needed to prove its usefulness for this purpose and for its use in routine clinical practice (31).

In our adjusted analyses, we observed that thrombosis is not only an important contributing factor to the pathogenesis of COVID-19 but also inflammation, which plays an important role, with both CRP and ferritin levels being associated with worse outcomes (Table 1). In multivariable analyses, with mortality (Table 2) or VTE (Table 3) as outcomes, D-dimer remained associated with these outcomes after adjusting for age, sex, comorbidities, illness severity, and CRP. Indeed, in a recent meta-analysis with a low level of heterogeneity (I^2^=48%, five studies, 594 patients, and no publication bias) patients with COVID-19 reached D-dimer levels up to 3,100 ng/mL FEU, even in the absence of any thrombotic event, with D-dimer showing significant results for worse prognosis (*p*<0.0001) (27).

Recent studies have used D-dimer levels as a prognostic enrichment factor, with conflicting evidence. The only published results so far are from the ACTION trial (12), which was neutral; however, the REMAP-CAP, ACTIV-4a, and ATTACC investigators found that, while critically ill patients do not benefit from full-dose anticoagulation (14), those with lower acuity may benefit from it (15). Other trials that did not use D-dimer as a biomarker also did not find any benefit from full-dose anticoagulation. Further studies are needed to evaluate how D-dimer levels can be used to guide anticoagulation strategies.

In COVID-19, we now largely know the important role of inflammation in the outcome of patients, as suggested by the beneficial effects of steroids and other immunomodulatory drugs. However, it is also known that UFH has important anti-inflammatory properties (28,29). In our manuscript, we observed a higher mortality in patients who used UFH, although this could be largely explained by its expected use in patients with acute kidney injury or chronic kidney disease.

Figure 2 shows when the analytes reach their highest levels during hospital admission and suggest that, if measured, it should be during the first week of admission and, when considered for a second measurement, it should also be in this first week of hospital admission to guide treatment decisions when alternative treatment approaches may be recommended.

We performed a cost analysis involving both surviving and deceased patients, as detailed in Table 5. Several demographic characteristics, such as sex and age, medical care facility used (emergency room, wards, or ICU), treatment costs (medication, transfusions, nutrition, hemodialysis, and mechanical ventilation), and tests (laboratory and radiological) can give us a picture of the high volume of the resources required and respective expenditures, particularly for deceased patients who were more ill. Considering the economic context and scarce resources, at the individual patient level, we should request tests that are essential for appropriate medical management, to estimate the prognosis, and to monitor complications.

### Implications for practice and future research

Our results strengthen the role of both inflammation and thrombosis as important characteristics of COVID-19 that merit consideration. Both D-dimer and CRP levels are independently associated with worse outcomes, that is, they are important prognostic factors (30). Taken together with the current literature, CRP has been included in prognostic models for COVID-19 (31,32), although D-dimer levels have not. Finally, both biomarkers have been studied as prognostic enrichment variables to guide treatment decisions (12,14,15,33). Although both tests may provide prognostic information, this does not necessarily mean that they lead to actionable information. Their daily routine utilization should be avoided, and they should be used in specific scenarios: D-dimer levels should be ordered to exclude VTE in patients with low to intermediate probability and when DIC is suspected. Its use as a prognostic factor and to guide treatment decisions is still under scrutiny and, if considered, should be performed on a single-dosing basis and not as a daily dosing routine. CRP, as a counterpart, could be measured during hospital admission for risk stratification through validated scores, such as the 4C score (31). In patients who are receiving steroids with no clinical improvement, repeated dosing could be considered and, if inflammation persists despite steroids, immune modulators, such as interleukin-6 inhibitors, can be considered (where available), or, alternatively, higher doses of steroids can be used (34).

## CONCLUSIONS

Elevated D-dimer and CRP levels in the first week of admission were associated with both higher hospital mortality and higher incidence of VTE and COVID-19 systemic inflammation in the adjusted analyses. Although D-dimer was more strongly associated with VTE occurrence, its discriminative ability was poor. Currently, their use outside of the usual indications for risk stratification or to guide treatment decisions should not be modified, and their routine utilization in the clinical context should be discouraged.

### HCFMUSP COVID-19 Study Group

Eloisa Bonfa, Edivaldo M. Utiyama, Aluisio C. Segurado, Beatriz Perondi, Anna Miethke-Morais, Amanda C. Montal, Leila Harima, Solange R. G. Fusco, Marjorie F. Silva, Marcelo C. Rocha, Izabel Marcilio, Izabel Cristina Rios, Fabiane Yumi Ogihara Kawano, Maria Amélia de Jesus, Ésper George Kallas, Carolina Carmo, Clarice Tanaka, Heraldo Possolo de Souza, Julio F. M. Marchini, Carlos Carvalho, Juliana C. Ferreira, Anna Sara Shafferman Levin, Maura Salaroli Oliveira, Thaís Guimarães, Carolina dos Santos Lázari, Ester Sabino, Marcello M. C. Magri, Tarcisio E. P. Barros-Filho, Maria Cristina Peres Braido Francisco, Silvia F. Costa. All investigators are from Hospital das Clinicas HCFMUSP, Faculdade de Medicina, Universidade de Sao Paulo, Sao Paulo, SP, BR.

## AUTHOR CONTRIBUTIONS

Gonçalves FAR, Bensen BAMP and Lima CA performed the literature search. Gonçalves FAR, Bensen BAMP, Fonseca LAM, Trindade EM, Sumita NM, Duarte AJS and Lichtenstein A designed the study. Gonçalves FAR, Bensen BAMP, Lima CA and Trindade EM collected the data. Gonçalves FAR, Bensen BAMP and Trindade EM analyzed the data. Gonçalves FAR, Bensen BAMP, Corá AP, Pereira AJR, Perazzio SF, Gouvea CP, Fonseca LAM, Trindade EM, Sumita NM, Duarte AJS and Lichtenstein A interpreted the data. Gonçalves FAR, Bensen BAMP, Corá AP, Pereira AJR, Perazzio SF, Gouvea CP, Fonseca LAM, Trindade EM, Sumita NM, Duarte AJS and Lichtenstein A wrote the manuscript.

## Figures and Tables

**Table 1:** Patients’ characteristics by primary outcome.

	Alive (N=1,761)	Deceased (N=717)	Total (N=2,478)	*p*-value
Sex (male)	911 (51.7%)	442 (61.6%)	1353 (54.6%)	<0.001 (1)
Age (years)	55.7 (54.9-56.4)	64.7 (63.6-65.7)	58.3 (57.6-58.9)	<0.001 (2)
Length of stay (days)	14.1 (13.6-14.7)	16.3 (15.5-17.1)	14.8 (14.3-15.2)	<0.001 (2)
Number of comorbidities	1.8 (1.7-1.9)	2.8 (2.7-2.9)	2.1 (2.0-2.1)	<0.001 (2)
Modified SOFA score	3.3 (3.2-3.5)	10.1 (9.8-10.3)	5.3 (5.1-5.5)	<0.001 (2)
Mechanical ventilation (yes)	388 (22.0%)	599 (83.5%)	987 (39.8%)	<0.001 (1)
Dialysis (yes)	106 (6.0%)	340 (47.4%)	446 (18.0%)	<0.001 (1)
Creatinine (mg/dL)	1.9 (1.8-2.1)	4.6 (4.4-4.8)	2.7 (2.6-2.8)	<0.001 (2)
Platelet count (x1000)	213 (209-218)	151 (145-157)	195 (191-199)	<0.001 (2)
D-dimer (altered)	494 (28.1%)	456 (63.6%)	950 (38.3%)	<0.001 (1)
C-reactive protein (altered)	488 (28.2%)	517 (75.3%)	1005 (41.6%)	<0.001 (1)
Ferritin (altered)	137 (22.5%)	105 (61.4%)	242 (31.0%)	<0.001 (1)

(1) Count (percentage) and Fisher’s exact test; (2) Mean (CI) and Wilcoxon rank sum test.

SOFA: Sequential Organ Failure Assessment.

**Table 2:** Crude and adjusted associations of D-dimer and C-reactive protein levels with hospital mortality.

	D-dimer Altered (4,000, +] *vs* Normal [0, 4,000]		C-reactive protein Altered (220, +] *vs* Normal [0, 220]	
	Odds ratio (95% CI)	*p*-value	Odds ratio (95% CI)	*p*-value
Crude	4.48 (3.73-5.39)	<0.001	7.73 (6.31-9.46)	<0.001
Model 1	4.27 (3.53-5.16)	<0.001	7.79 (6.30-9.62)	<0.001
Model 2	3.94 (3.24-4.80)	<0.001	7.09 (5.71-8.80)	<0.001
Model 3	2.50 (2.01-3.09)	<0.001	4.46 (3.53-5.62)	<0.001
Model 4	1.97 (1.56-2.47)	<0.001	3.93 (3.10-4.99)	<0.001
Model 5	2.01 (1.59-2.53)	<0.001	3.98 (3.14-5.06)	<0.001

Model 1: adjusted for age and sex.

Model 2: adjusted for model 1+number of comorbidities.

Model 3: adjusted for model 2+admission modified SOFA score.

Model 4: adjusted for model 3+D-dimer or C-reactive protein included in the model.

Model 5: adjusted for model 4 + imaging-confirmed venous thromboembolism.

SOFA: Sequential Organ Failure Assessment.

**Table 3:** Crude and adjusted associations of D-dimer levels and C-reactive protein levels with imaging-confirmed venous thromboembolism.

	D-dimer Altered (4,000, +] *vs* Normal [0, 4,000]		C-reactive protein Altered (220, +] *vs* Normal [0, 220]	
	Odds ratio (95% CI)	*p*-value	Odds ratio (95% CI)	*p*-value
Crude	3.96 (2.92-5.36)	<0.001	2.71 (2.02-3.65)	<0.001
Model 1	4.00 (2.94-5.43)	<0.001	2.71 (2.01-3.66)	<0.001
Model 2	3.93 (2.88-5.37)	<0.001	2.65 (1.96-3.60)	<0.001
Model 3	3.82 (2.76-5.29)	<0.001	2.50 (1.80-3.47)	<0.001
Model 4	3.26 (2.33-4.56)	<0.001	1.92 (1.37-2.69)	<0.001

Model 1: adjusted for age and sex.

Model 2: adjusted for model 1+number of comorbidities.

Model 3: adjusted for model 2+admission modified SOFA score.

Model 4: adjusted for model 3+D-dimer or C-reactive protein included in the model.

SOFA: Sequential Organ Failure Assessment.

**Table 4:** Absolute distribution of 2,478 patients by vital status and levels of anticoagulants.

Outcome		D-Dimer Altered (N=950)	D-Dimer Normal (N=1,528)	Ferritin Altered (N=237)	Ferritin Normal (N=543)	C-reactive protein Altered (N=1,005)	C-reactive protein Normal (N=1,410)	*p*-value
Alive	Anticoagulants (levels)							<0.001 (1)
	- No or eventually use	54	236	17	58	47	223	
	- LMWH therapeutic	35 (8.0%)	18 (1.7%)	11 (9.3%)	19 (4.6%)	35 (7.9%)	18 (1.8%)	
	- LMWH thromboprophylaxis	69 (15.7%)	56 (5.4%)	20 (16.9%)	47 (11.3%)	65 (14.7%)	60 (5.9%)	
	- UFH therapeutic in AKI	9 (2.0%)	0 (0.0%)	2 (1.7%)	5 (1.2%)	8 (1.8%)	1 (0.1%)	
	- UFH therapeutic in CKD	102 (23.2%)	114 (11.1%)	17 (14.4%)	65 (15.6%)	80 (18.1%)	135 (13.3%)	
	- UFH thromboprophylaxis	225 (51.1%)	843 (81.8%)	68 (57.6%)	280 (67.3%)	253 (57.4%)	803 (79.0%)	
Deceased	Anticoagulants (levels)							0.106 (1)
	- No or eventually use	113	71	20	8	114	60	
	- LMWH therapeutic	65 (19.0%)	22 (11.6%)	20 (24.4%)	13 (21.3%)	72 (17.9%)	13 (11.8%)	
	- LMWH thromboprophylaxis	102 (29.7%)	43 (22.6%)	26 (31.7%)	12 (19.7%)	112 (27.8%)	30 (27.3%)	
	- UFH therapeutic in AKI	12 (3.5%)	5 (2.6%)	0 (0.0%)	2 (3.3%)	14 (3.5%)	2 (1.8%)	
	- UFH therapeutic in CKD	24 (7.0%)	18 (9.5%)	6 (7.3%)	4 (6.6%)	35 (8.7%)	6 (5.5%)	
	- UFH thromboprophylaxis	140 (40.8%)	102 (53.7%)	30 (36.6%)	30 (49.2%)	170 (42.2%)	59 (53.6%)	

(1) Count (percentage) and Fisher’s exact test.

UFH: Unfractioned heparin; LMWH: Low-molecular weight heparin; AKI: acute kidney injury; CKD: chronic kidney disease.

**Table 5:** Cost components.

	No VTE and Alive (N=1,628)	No VTE and Deceased (N=640)	VTE and Alive (N=133)	VTE and Deceased (N=77)	Total (N=2,478)	*p*-value
Total length of stay	13 (11)	16 (11)	26 (16)	22 (11)	15 (11)	<0.001 (1)
Total cost	$7,444 ($9,604)	$17,637 ($16,114)	$18,083 ($16,859)	$25,099 ($15,260)	$11,196 ($13,356)	<0.001 (1)
Emergency department	$62 ($65)	$68 ($50)	$67 ($50)	$66 ($62)	$64 ($61)	<0.001 (1)
Intensive care unit	$8,970 ($7,570)	$11,207 ($8,391)	$13,604 ($11,622)	$15,929 ($9,014)	$10,632 ($8,540)	<0.001 (1)
Hospital wards	$2,169 ($1,679)	$942 ($1,210)	$3,425 ($2,323)	$1,217 ($1,290)	$2,031 ($1,754)	<0.001 (1)
Medical staff	$175 ($165)	$143 ($150)	$273 ($228)	$177 ($175)	$173 ($168)	<0.001 (1)
Drugs	$537 ($1,089)	$1,992 ($2,702)	$1,602 ($2,219)	$3,396 ($3,709)	$1,060 ($1,986)	<0.001 (1)
Laboratory tests	$533 ($592)	$1,213 ($964)	$1,115 ($961)	$1,875 ($1,266)	$782 ($837)	<0.001 (1)
Radiologic exams	$166 ($158)	$176 ($174)	$309 ($254)	$315 ($268)	$182 ($179)	<0.001 (1)
Blood components	$1,946 ($7,497)	$2,280 ($12,913)	$1,440 ($3,238)	$706 ($1,192)	$1,949 ($10,005)	0.043 (1)
Nutrition	$496 ($306)	$350 ($224)	$679 ($335)	$458 ($239)	$433 ($282)	<0.001 (1)
Hemodialysis	$100 ($26)	$235 ($700)	$127 ($49)	$164 ($271)	$191 ($562)	0.021 (1)
Mechanical ventilation	$1,589 ($1,391)	$2,516 ($1,870)	$2,325 ($2,051)	$3,281 ($1,794)	$2,232 ($1,798)	<0.001 (1)

(1) Mean (SD) and Kruskal-Wallis rank sum test.

Note: VTE: Venous thromboembolism.
